# Supplementary figures and images for: Peptides derived from the SARS-CoV-2 receptor binding motif bind to ACE2 but do not block ACE2-mediated host cell entry or pro-inflammatory cytokine induction
Source: PLoS One. 2021 Nov 18;16(11):e0260283. doi: 10.1371/journal.pone.0260283 (PMC8601423; doi:10.1371/journal.pone.0260283)

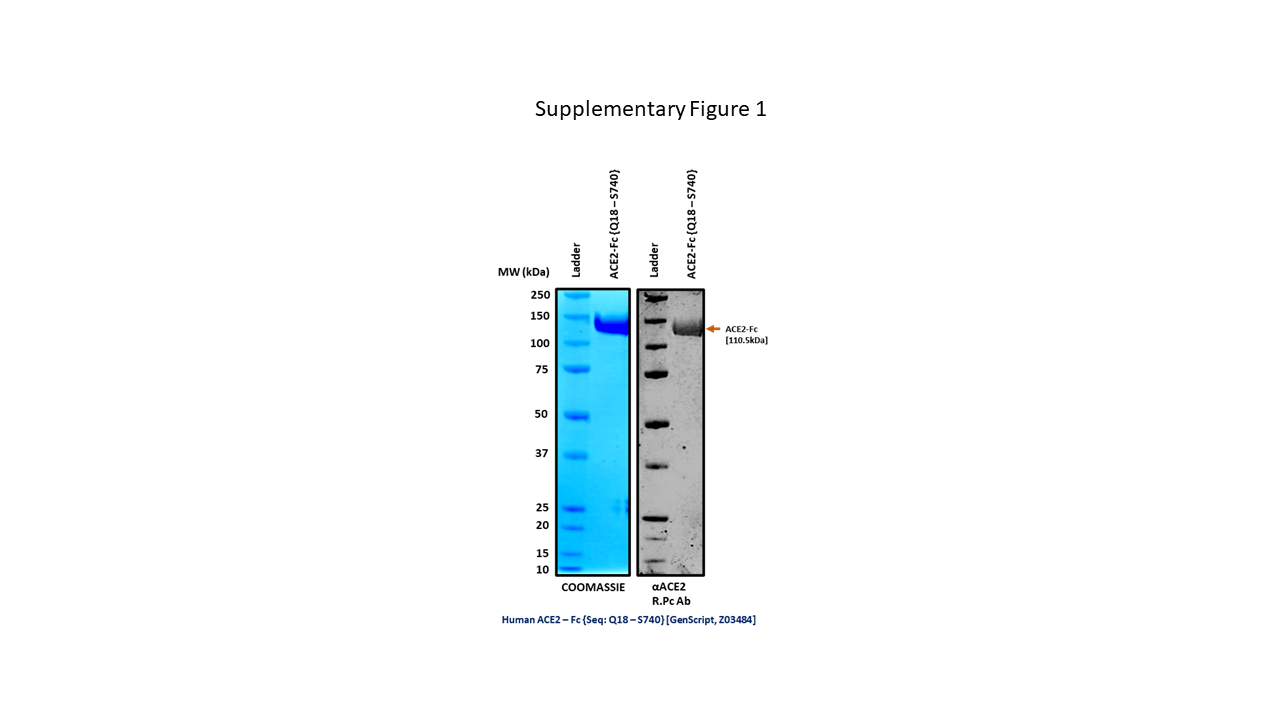

Supplement: S1 Fig — Human purified recombinant ACE2-Fc (Q18-S740; GenScript), detected via SDS-PAGE by (left) Coomassie staining and (right) western immunoblotting with an αACE2 rabbit polyclonal antibody. Lane 1: Ladder, Lane 2: ACE2-Fc protein. ACE2-Fc protein detected at expected molecular weight (~110.5kDa). (TIF) [file pone.0260283.s001.tif]

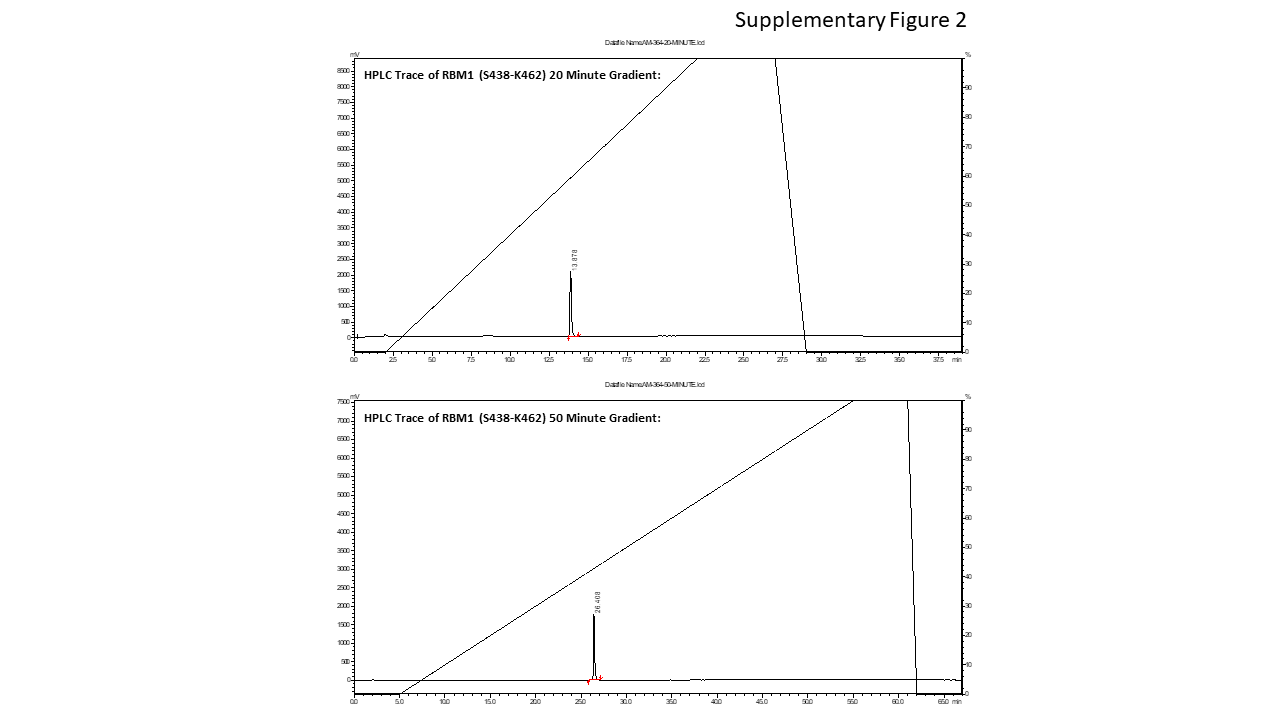

Supplement: S2 Fig — (TIF) [file pone.0260283.s002.tif]

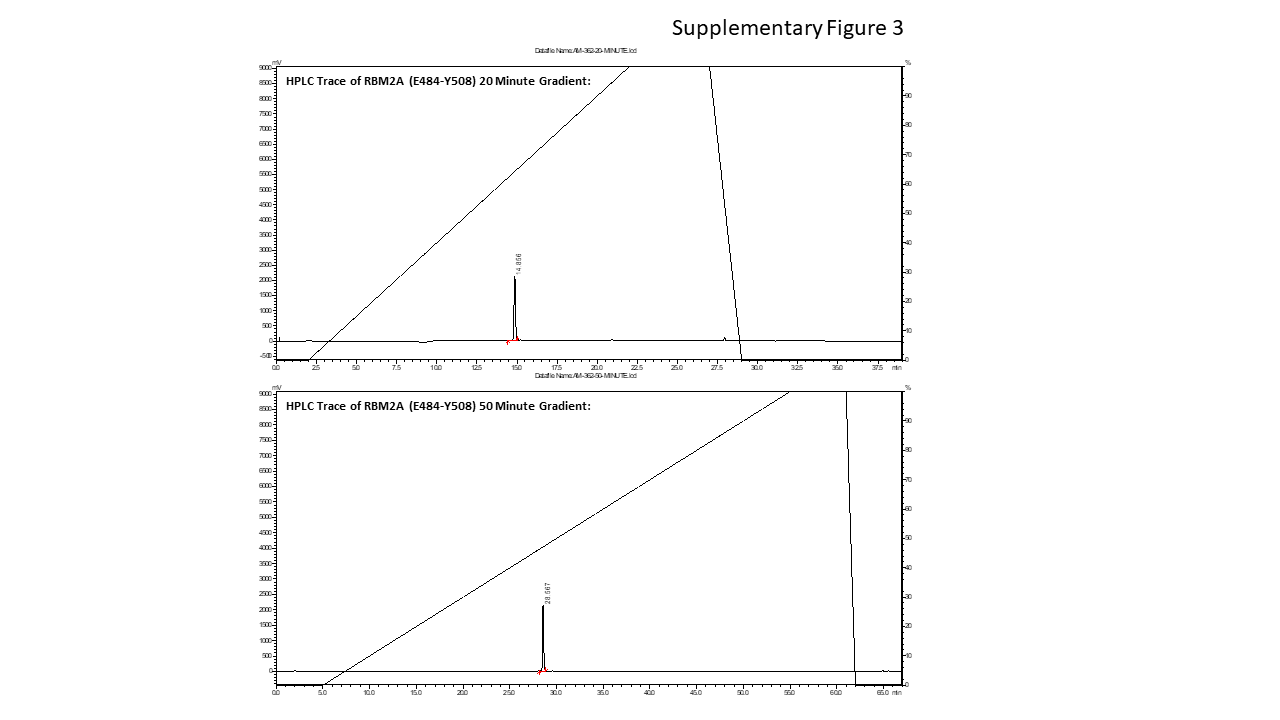

Supplement: S3 Fig — (TIF) [file pone.0260283.s003.tif]

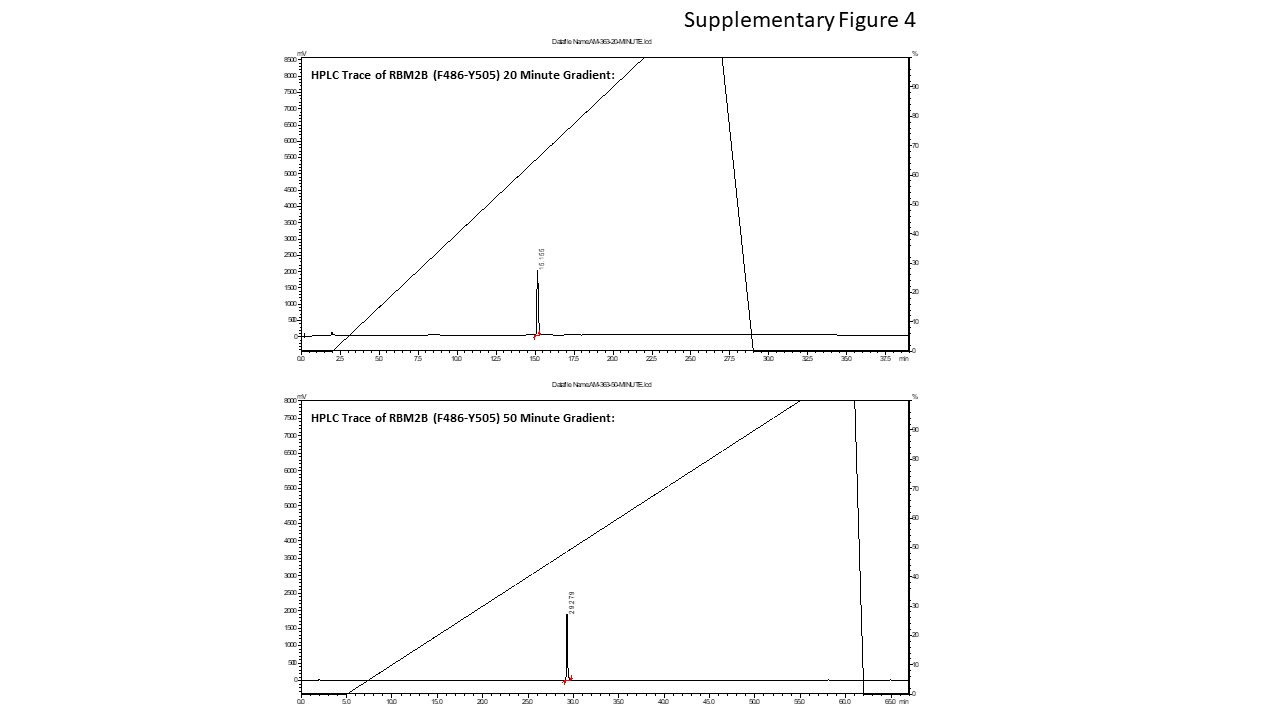

Supplement: S4 Fig — (TIF) [file pone.0260283.s004.tif]

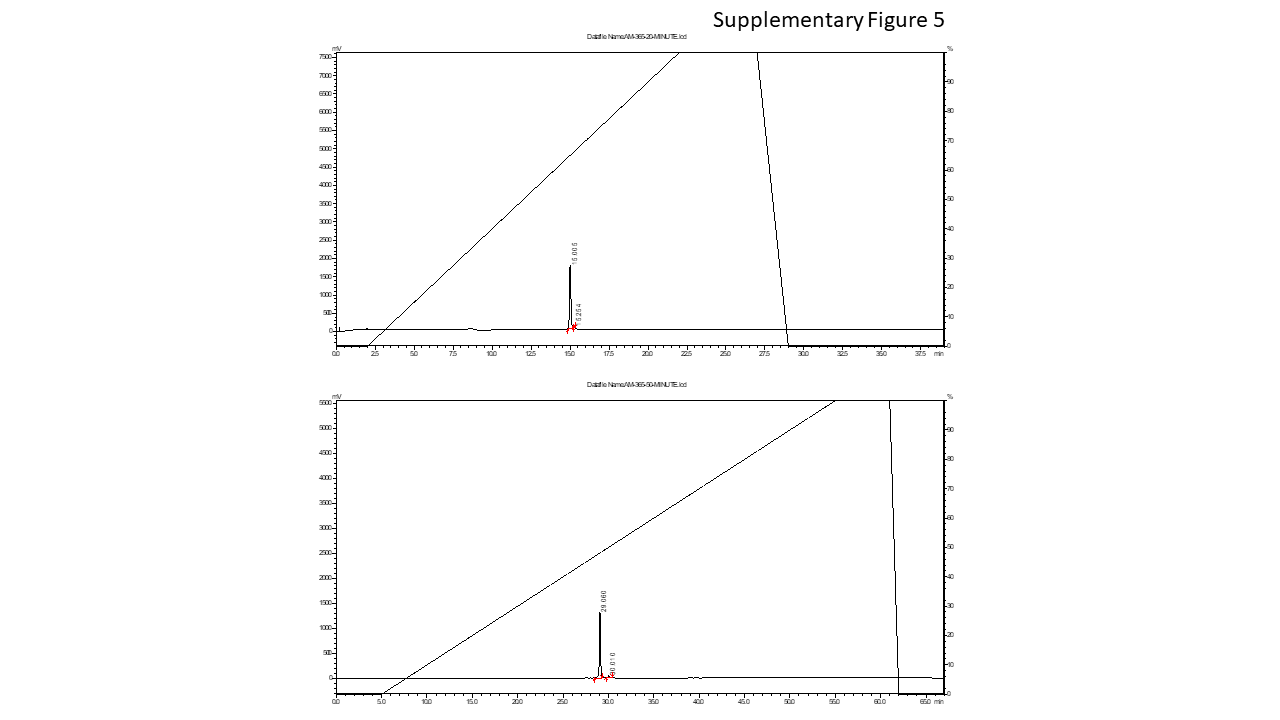

Supplement: S5 Fig — (TIF) [file pone.0260283.s005.tif]

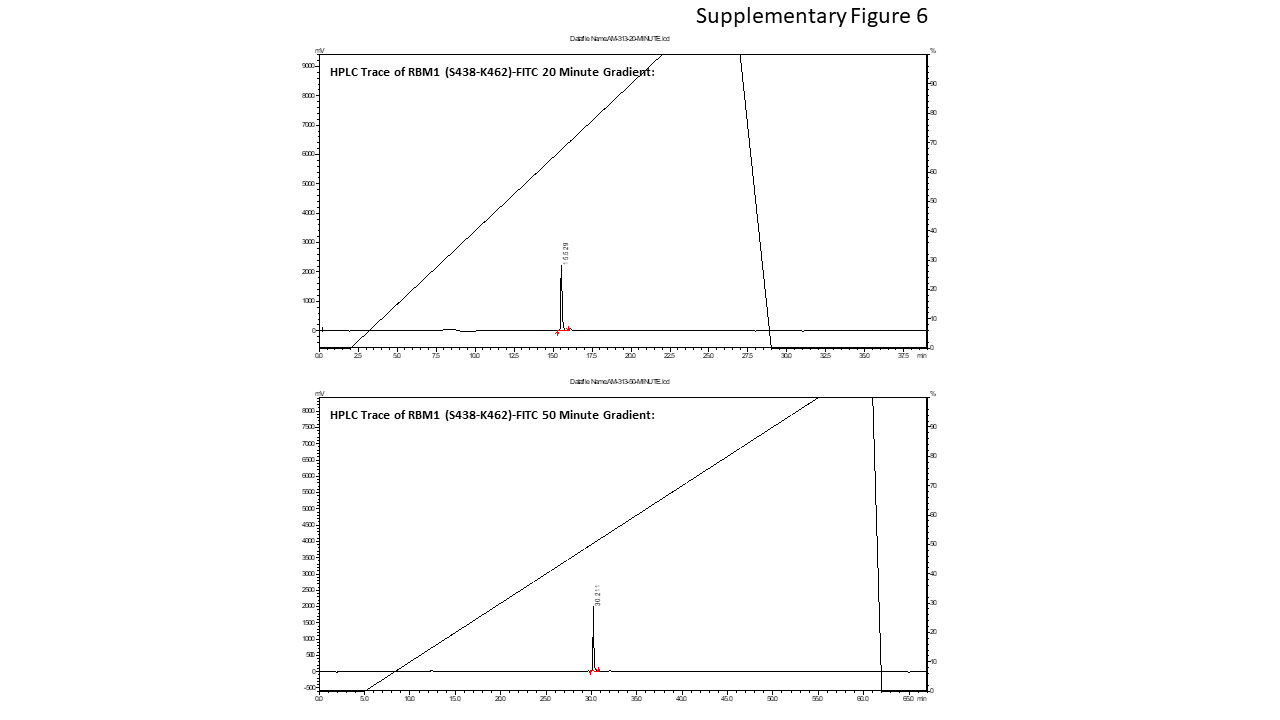

Supplement: S6 Fig — (TIF) [file pone.0260283.s006.tif]

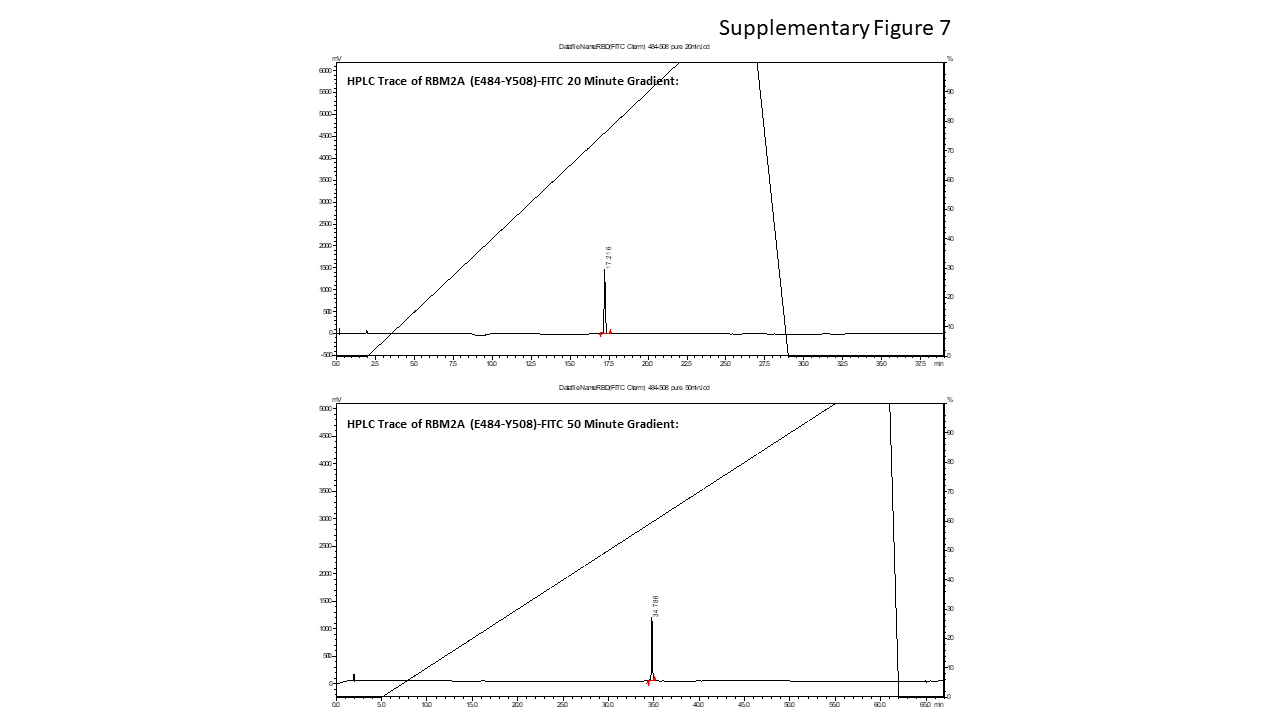

Supplement: S7 Fig — (TIF) [file pone.0260283.s007.tif]

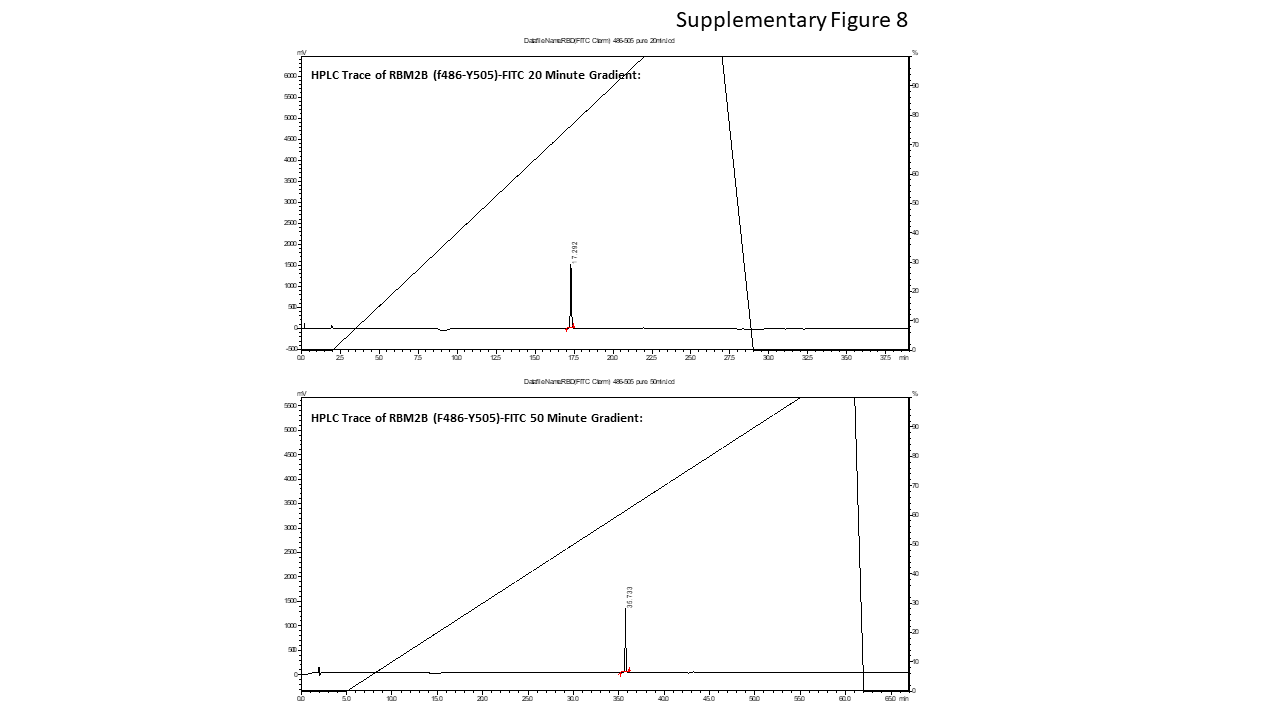

Supplement: S8 Fig — (TIF) [file pone.0260283.s008.tif]

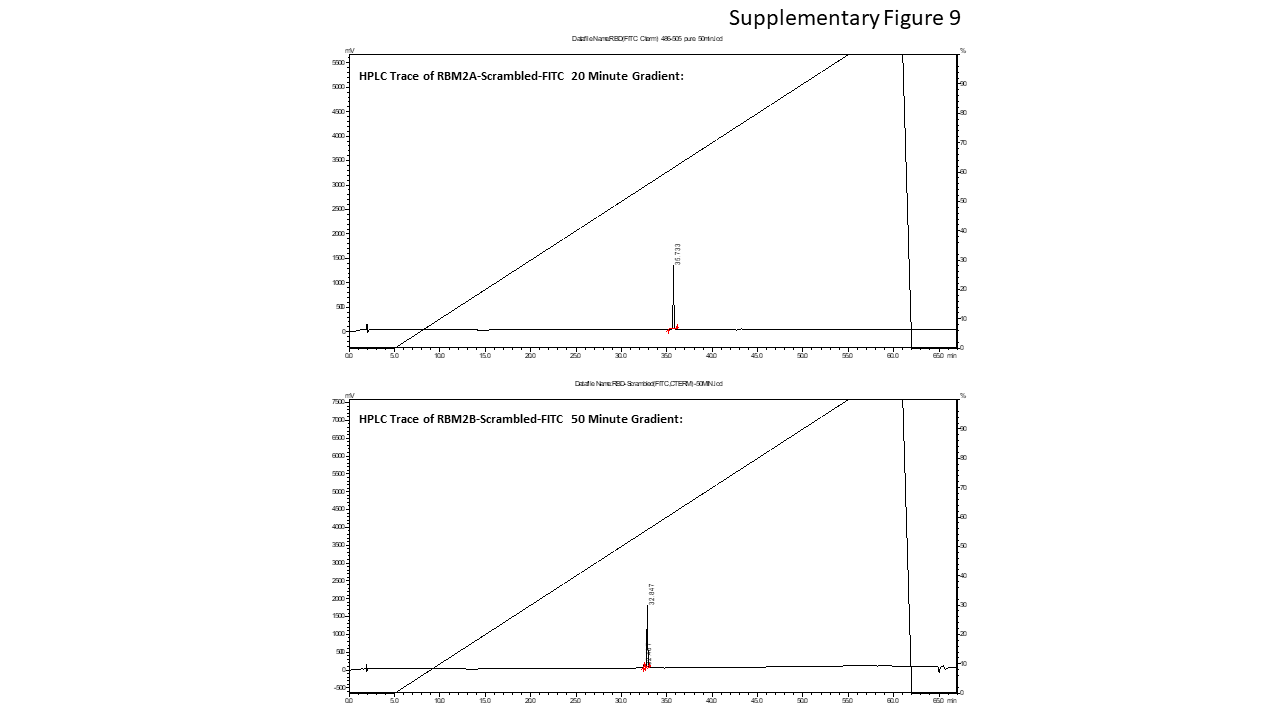

Supplement: S9 Fig — (TIF) [file pone.0260283.s009.tif]
