# Supplementary material for: Peptides derived from the SARS-CoV-2 receptor binding motif bind to ACE2 but do not block ACE2-mediated host cell entry or pro-inflammatory cytokine induction
Source: PLoS One. 2021 Nov 18;16(11):e0260283. doi: 10.1371/journal.pone.0260283 (PMC8601423; doi:10.1371/journal.pone.0260283)

Raw_Uncropped (full) immunoblots: RE: Figure 5A in Manuscript

Each immunoblot has same layout:
Lane 1: Ladder
Lane 2: A549-Empty
Lane 3: A549-Empty + S-RBD (100nM)
Lane 4: A549-ACE2
Lane 5: A549-ACE2 + S-RBD (100nM) + Vehicle
Lane 6: A549-ACE2 + S-RBD (100nM) + RBM2A-Sc (5uM)
Lane 7: A549-ACE2 + S-RBD (100nM) + RBM2A (5uM)
Lane 8: A549-ACE2 + S-RBD (100nM) + RBM2B (5uM)
Lane 9: A549-ACE2 + S-RBD (100nM) + RBM1 (5uM)
Lane 10: A549-ACE2 + S-RBD (100nM) + RBM1 (5uM) + RBM2A (5uM)

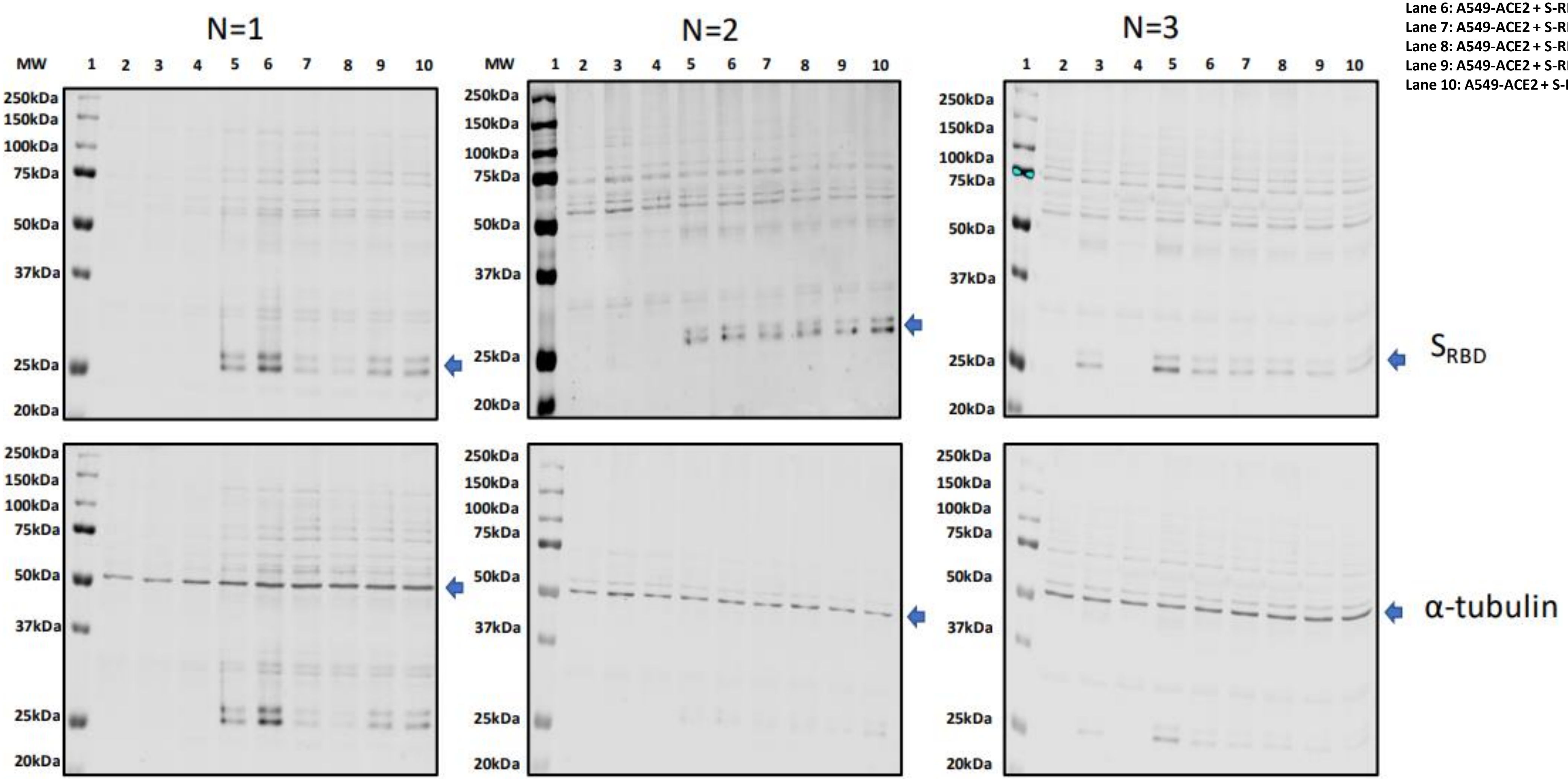

Supplement: S1 Raw image — (PDF) [file pone.0260283.s010.pdf]
