# Supplementary material for: Peptides derived from the SARS-CoV-2 receptor binding motif bind to ACE2 but do not block ACE2-mediated host cell entry or pro-inflammatory cytokine induction
Source: PLoS One. 2021 Nov 18;16(11):e0260283. doi: 10.1371/journal.pone.0260283 (PMC8601423; doi:10.1371/journal.pone.0260283)
Supplement: Attachment — Submitted filename: Response to Reviewers.docx [file pone.0260283.s017.docx]

**PLOS One Rebuttal Letter**

**PONE-D-21-28336**

Peptides derived from the SARS-CoV-2 Receptor Binding Motif bind to ACE2 but do not block ACE2-mediated host cell entry or pro-inflammatory cytokine induction.

Corresponding Author: Dr. Connor M. Blair ([connor.blair@glasgow.ac.uk](mailto:connor.blair@glasgow.ac.uk))

(0) References have been updated so that they run in an appropriate numerical/sequential order in text and in reference list – as requested. None of the references included have been retracted. However, reference 37 (Yang, et al. 2021 Science) has had a ‘correction’ published: {correction: Yang L, Xie X, Tu Z, Fu J, Xu D, Zhou Y. The signal pathways and treatment of cytokine storm in COVID-19. Signal Transduct Target Ther. 2021;6(1):326}. Correction was published Aug 31^st^ 2021 ( ~7 weeks after original publication). Original publication has been updated on Nature Publishing Groups webpage and therefore the original citation marked reference 37 is an up-to-date draft and will direct you to the intended/appropriate review article. I hope this is appropriate.
(1) Figures have been re-uploaded following analysis on PACE.
(2) Manuscript Style Requirements: submitted manuscript is .docx file, Calibri (body), double spaced, with abbreviations stated early on in text. Vancouver style was used for referencing. Each figure has been attached as a separate/individual file. Please let me know specifically if there is any other aspect of style in which I am not meeting standards.

(3) Amended Statements:

a. Funding Statement should read:
   *‘CMB, MR, GT and OJ are supported through the WT ISSF COVID Response Award (204820/Z/16/Z). AM is supported through the EPSRC (Research Project Grant EP/N034260/2) and CM is supported through Dstl (DSTLX-1000141308). DCM thanks the EPSRC for a studentship (EP/N509668/1 and EP/R513222/1). RMT is supported through a BHF Chair award (CH/4/29762). ACM is supported by a University of Glasgow Walton Fellowship. DB and IH were supported by the Medical Research Council (MC UU 12014/7).’*

(4) & (5) Minimal data files have been updated in the supplementary files, including:
    a. Minimal data file relating to fluorescent polarisation experiments:
       name: ‘*Fig 4_Fluorescent Polarisation_Min Data.xlsx’*
    b. Minimal data file relating to western blotting experiments (including uncropped IBs):
       name: *‘Fig 5A_WB Internalisation Assay_Min Data.pzfx’* AND *‘Fig 5A_WB Internalisation Assay_ Uncropped WBs.pdf’*
    c. Minimal data file relating to luminescence experiments:
       name: *‘Fig 5B_N-Luc Internalisation Assay_Min Data.pzfx’*
    d. Minimal data file relating to pseudovirus experiments:
       name: *‘Fig 6_Spike PsV Inhibition Assay_Data.xlsx’*
    e. Minimal data file relating to inflammatory marker experiments:
       name: *‘Fig 7_CMEC Inflammation Marker_Min Data.xlsx’*
(6) Tables in manuscript: I have not included table 2 within the original main body of the manuscript submitted. Instead, table 2 was found within the combined figure file that was attached/submitted. As per request, I have removed Table 2 from the original combined Figure File and have now inserted into the main body of the manuscript at the appropriate point. Thus, both Table 1 and 2 are within main body of manuscript only.
    The table that is included in Figure 1 is part of Figure 1 and I therefore request that it remains this way. I hope this meets the requirements.
    Note that all figures (including supplementary) have been uploaded as separate .TIF files as per request. I have also included a PDF file with ALL figures. Please select whichever comes out with highest resolution.
(7) Response to Reviewer 1:
    a. Regarding comments RE: **Lines 81-85**…
       I have made amendments to acknowledge the fact that SARS-CoV-2 spike protein binds ACE2 with significantly higher affinity than SARS-CoV-1 spike protein. I have also acknowledged that the binding affinity of our peptides with ACE2 is needed to be significantly higher than the SAR-CoV-1 RBM Hexapeptide if to be competitive against the spike protein. (Wrapp, et al 2020) reference has also been added in. I hope this will suffice:
       *“Previous work utilising a SARS-CoV-1 spike RBM derived hexapeptide (Y438-K-Y-R-Y-L443) demonstrated a modest ability to directly bind ACE2 (K_d_ = 46 µM) and inhibit coronavirus NL63 cell entry and subsequent viral replication* ***^19^****. Although this peptide was not tested against SARS-CoV-1 viral entry, these findings highlight the possibility for a SARS-CoV-2 RBM-derived peptide to represent a potentially promising approach to the development of novel antiviral decoy peptides. However, as the spike protein corresponding to SARS-CoV-2 has been shown to bind ACE2 10 times more tightly than SARS-CoV-1, the binding affinity of SARS-CoV-2 derived peptide(s) will need to be significantly higher in order to be competitive against the spike protein* ***^33^”***
    b. Regarding comments RE: **rational behind selecting which region on SARS**…
       I have added further explanation as to why we selected these two peptides as high confidence starting points for the development of RBD – ACE2 PPI decoy peptides (Lines 275-290). Selection is initially derived from data that exists within the public domain – i.e. existing solved co-crystal structures and related publications that have already carried out extensive analysis. I have referenced these in text appropriately.
       As analysis regarding contact residues between SARS-CoV-2 RBM and ACE2 has already been extensively investigated and published (references already included in manuscript), I have not included data in Figure 1 that details my own analysis as it would be directly replicating data that is already available and therefore add no novel value to the figure/study. As such, Figure 1 remains the same – highlighting the known contact residues of SARS-CoV-2 RBM that directly interact with ACE2. I have also highlighted these residues (in red) in a published (solved) co-crystal structure of the SARS-CoV-2 RBD – ACE2 protein-protein interaction for qualitative purposes (visualisation). This structure can be found on PDB and the protein structure I have used in the figure was generated using PyMol software. I hope this suffices.
    c. Regarding comments RE: **showing luminescence experimental set up is sound**…
       Having discussed this with Dr Brian Hudson and Dr Mario Rossie (the two researchers responsible for developing and carrying out the luciferase assay), they have forwarded the following response to your comment(s):
       ‘We appreciate the reviewers’ comment, however we would also like to highlight that these internalization approaches based on the pH dependence of the NanoLuc activity have already been tested extensively by the scientific community. These tools have in fact been considered well established, valuable approaches for quantification of cell membrane protein internalization, especially in HEK293 cell lines. In the resubmitted version of the paper, you will find two new reference articles showing nano luciferase as a sensitive bioluminescent reporter to measure quantitatively the internalization of cell membrane receptors.
       “Quantitative measurement of cell membrane receptor internalization by the nanoluciferase reporter: Using the G protein-coupled receptor RXFP3 as model. Y. Liu et al. / Biochimica et Biophysica Acta 1848 (2015) 688–694”, “A luminescent assay for real-time measurements of receptor endocytosis in living cells. M.B. Robers et al. / Analytical Biochemistry 489 (2015) 1e82“’
       I hope this amendment/response will suffice.
(8) Response to Reviewer 2:
    a. Sequences added to abstract as requested.
    b. Although we did not generate any data regarding point mutations within our peptides that would confer higher ACE2 binding affinity, I have included a small section in the updated tracked changed document (Lines 406-410) briefly discussing the possibility of selecting for already known RBD point mutations that could lead to higher ACE2 binding affinity of our selected RBM peptides. Due to grant funding/duration coming to an end, we are unable to carry out further experiments relating to mutations. I hope this statement + relevant addition of references will suffice.
       *“Furthermore, analysis of global genome sequencing datasets detailing identified mutations within the receptor binding domain have identified variants that possess higher ACE2 binding affinity (e.g. Y453F and N501Y) compared with the original Wuhan-1 strain* ***^33,34^****. Resultingly, these mutations could be leveraged in future efforts aimed at selecting peptide sequences that possess higher ACE2 binding affinity.”*
